# A novel MRI analysis for assessment of microvascular vasomodulation in low-perfusion skeletal muscle

**DOI:** 10.1038/s41598-020-61682-z

**Published:** 2020-03-13

**Authors:** Eric Zakher, Tameshwar Ganesh, Hai-Ling Margaret Cheng

**Affiliations:** 1 The Edward S. Rogers Sr. Department of Electrical & Computer Engineering, University of Toronto, Toronto, Canada; 2 Ted Rogers Centre for Heart Research, Translational Biology & Engineering Program, Toronto, Canada; 3 Institute of Biomaterials & Biomedical Engineering, University of Toronto, Toronto, Canada; 4 Heart & Stroke/Richard Lewar Centre of Excellence in Cardiovascular Research, Toronto, Canada; 5 Ontario Institute for Regenerative Medicine, Toronto, Canada

**Keywords:** Vasodilation, Biomedical engineering, Biological physics

## Abstract

Compromised microvascular reactivity underlies many conditions and injuries, but its assessment remains difficult, particularly in low perfusion tissues. In this paper, we develop a new mathematical model for the assessment of vasomodulation in low perfusion settings. A first-order model was developed to approximate changes in *T*_1_ relaxation times as a result of vasomodulation. Healthy adult rats (*N* = 6) were imaged on a 3-Tesla clinical MRI scanner, and vasoactive response was probed on gadofosveset using hypercapnic gases at 20% and 5% CO_2_ to induce vasoconstriction and vasodilation, respectively. MRI included dynamic 3D *T*_1_ mapping and *T*_1_-weighted images during gas challenge; heart rate was continuously monitored. Laser Doppler perfusion measurements were performed to corroborate MRI findings. The model was able to identify hypercapnia-mediated vasoconstriction and vasodilation through the partial derivative $\frac{\partial {T}_{1}}{\partial t}$. MRI on animals revealed gradual vasoconstriction in the skeletal muscle bed in response to 20% CO_2_ followed by gradual vasodilation on transitioning to 5% CO_2_. These trends were confirmed on laser Doppler perfusion measurements. Our new mathematical model has the potential for detecting microvascular dysfunction that manifests in the early stages across multiple metabolic and ischemic pathologies.

## Introduction

The microvasculature is a roadway connecting the cells in our body and is the only site where nutrient and waste exchange occurs. It also plays a critical role in maintaining blood pressure and altering local blood supply in response to changing metabolic demands. When the microvasculature becomes dysfunctional, as seen in diabetes and hypertension, this ability to adjust blood supply is compromised. In fact, ample evidence points to microvascular dysfunction as one of the earliest yet undetectable symptoms across a spectrum of conditions, including diabetes, hypertension, obesity, dementia, and coronary artery disease^1–3^ – undetectable because a diagnostic technique does not exist for this purpose. Because the vasculature is a connected entity, microvascular pathology is usually not confined to one anatomical location but extends to other tissue beds. Skeletal muscle is one tissue where microvessels are affected across numerous conditions, such as metabolic syndromes (e.g. diabetes, obesity) and neuropathy^4^, to name a few. In diabetic patients, for example, exercise intolerance stems from inadequate blood supply to the leg muscle^5^. Identifying skeletal muscle microvascular pathology is, therefore, a potential target for early disease detection.

Imaging muscle microvascular function, however, is challenging. The skeletal muscle bed has a much lower signal compared to other soft tissues when an imaging contrast agent is injected intravenously to fill blood vessels. The lower signal stems from the relatively low perfusion of skeletal muscle, which, at 0.43% of the total cardiac output relative to organ weight, is the lowest of all organs^6^. As such, it is very difficult to sensitively detect vasomodulation-induced dynamic changes in microvascular volume. In a recent innovation described by Ganesh *et al*.^7^, a MRI technique was demonstrated for measuring the dynamics of microvessel modulation in deep organs. Their technique is unique compared to existing clinical methods to assess the microvasculature, because it can isolate strictly changes in microvessel volume and can, therefore, truly measure vasomodulation. In contrast, methods such as laser Doppler flowmetry^8^, notwithstanding limited depth penetration, do not specifically measure vasomodulation, because its measurement of perfusion also reflects changes in heart rate. Techniques such as flow-mediated dilation^9^ are even less comparable, because those methods assess flow in large arteries, typically the brachial artery, and not in small vessels. However, in its current form, the approach described in ref. ^7^ is relatively insensitive to vasomodulation in low perfusion tissue such as skeletal muscle; we need to use the approach as a starting point to build the desired capability.

In this paper, we propose a new analytical method that allows the extraction of microvessel vasomodulation in low perfusion tissue. This method is shown to be sensitive to both vasoconstriction and vasodilation in the microvascular bed of skeletal muscle.

## Theory

The intravenous administration of a ${T}_{1}$ contrast agent is used to impart higher signal intensity to tissue; this is achieved through the reduction of ${T}_{1}$ relaxation times of water protons in close proximity to the agent and through the use of ${T}_{1}$-weighted sequences. Tissues that are more richly vascularized will undergo relatively greater contrast enhancement. If the contrast agent remains intravascular, essentially only the blood-pool water protons will experience enhanced relaxation effects. In this scenario, any signal changes in a voxel can be assumed to arise primarily from changes in the vascular volume fraction in that voxel, because this is the only space where the contrast agent resides. In other words, if the vascular volume increases through vasodilation or decreases through vasoconstriction, the ${T}_{1}$ relaxation effect will increase or decrease, respectively – the ${T}_{1}$ relaxation time is effectively dominated by the blood volume fraction.

To appreciate how the blood volume fraction in a voxel is related to the overall ${T}_{1}$, let us examine Eq. (1), which states that the longitudinal relaxation rate (1/${T}_{1}$) is proportional to the concentration of contrast agent in a homogeneous system:

1 $$\frac{1}{{{\boldsymbol{T}}}_{1}}=\frac{1}{{{\boldsymbol{T}}}_{10}}+{{\boldsymbol{r}}}_{1}{\boldsymbol{C}}$$

where $1/{T}_{10}$ is the relaxation rate in the absence of contrast, $C$ is the time-dependent concentration of the contrast agent, and ${r}_{1}$ is a constant for a given contrast agent known as the relaxivity. Since the contrast agent is confined to the vascular space, its concentration in an imaging voxel is simply the contrast concentration in blood (${C}_{b}$) weighted by the fractional blood volume (${v}_{b}$). We can rewrite Eq. (1) as follows:

2 $$\frac{1}{{T}_{1}}=\frac{1}{{T}_{10}}+{r}_{1}{C}_{b}{v}_{b}$$

The evolution of ${T}_{1}$ with time will be influenced by renal excretion of the contrast agent and any vasomodulation (i.e. changes in ${v}_{b}$) that may be present. To understand this temporal evolution, we take the partial derivative of Eq. (2) with respect to time:

3 $$\frac{\partial }{\partial {\boldsymbol{t}}}\left(\frac{1}{{{\boldsymbol{T}}}_{1}}\right)=\frac{\partial }{\partial {\boldsymbol{t}}}\left(\frac{1}{{{\boldsymbol{T}}}_{10}}+{{\boldsymbol{r}}}_{1}{{\boldsymbol{C}}}_{{\boldsymbol{b}}}{{\boldsymbol{v}}}_{{\boldsymbol{b}}}\right)$$

We solve Eq. (3) by taking the quotient rule on the left hand side and the product rule on the right hand side. For practical purposes, we can assume $\,{T}_{10}$ is constant with time, as the longitudinal relaxation of tissue with undoped blood changes minimally with blood volume fluctuations^10^. Since both ${C}_{b}$ and ${v}_{b}$ are time-dependent, the result is:

4 $$\frac{\partial {T}_{1}}{\partial t}=r{T}_{1}^{2}\left(-\frac{\partial {C}_{b}}{\partial t}{v}_{b}-{C}_{b}\frac{\partial {v}_{b}}{\partial t}\right)$$

Assuming that the renal elimination rate is a positive-valued constant $K=-\,\frac{\partial {C}_{b}}{\partial t}$ in Eq. (4), we arrive at the following final equation to analyze vasomodulation in low perfusion tissue:

5 $$\frac{\partial {{\boldsymbol{T}}}_{1}}{\partial {\boldsymbol{t}}}=\,{\boldsymbol{r}}{{\boldsymbol{T}}}_{1}^{2}\left({\boldsymbol{K}}{{\boldsymbol{v}}}_{{\boldsymbol{b}}}-{{\boldsymbol{C}}}_{{\boldsymbol{b}}}\frac{\partial {{\boldsymbol{v}}}_{{\boldsymbol{b}}}}{\partial {\boldsymbol{t}}}\right)$$

Equation (5) enables us to understand what the partial derivative $\frac{\partial {T}_{1}}{\partial {\rm{t}}}$ signifies physiologically. Table 1 describes the main scenarios that may arise *in vivo*. We consider each scenario in the following.

**Table 1:** Characteristics of the partial derivative in relation to physiological status.

Scenario	Renal Clearance	Vasomodulation	$\frac{\partial {{\boldsymbol{T}}}_{1}}{\partial {\boldsymbol{t}}}$	
			Sign	Temporal characteristics
K = 0, $\frac{\partial {v}_{b}}{\partial t}$> 0	No	Vasodilation	Negative	N/A
K = 0, $\frac{\partial {v}_{b}}{\partial t}$ < 0	No	Vasoconstriction	Positive	N/A
K = constant, $\frac{\partial {v}_{b}}{\partial t}$ = 0	Yes	None	Positive	N/A
K = constant, $\frac{\partial {v}_{b}}{\partial t}$ > 0	Yes	Vasodilation	Negative for rapid changes in ${v}_{b}$	Increasing trend
K = constant, $\frac{\partial {v}_{b}}{\partial t}$ < 0	Yes	Vasoconstriction	Positive	Decreasing trend

Scenarios 1 and 2 represent the simplest case where the renal elimination rate constant *K* is zero. Although this situation never occurs in practice, it represents the extreme situation where the contrast agent does not leave the body and the blood concentration remains constant. Under these ideal circumstances, positive and negative $\frac{\partial {T}_{1}}{\partial t}$ values signify vasoconstriction and vasodilation, respectively, and the magnitude of $\frac{\partial {T}_{1}}{\partial t}$ describes how fast vasomodulation occurs.

Scenarios 3 to 5 represent the more realistic situation where renal elimination is present. In the absence of vasomodulation (Scenario 3), $\frac{\partial {T}_{1}}{\partial t}$ takes on a positive value, and we do not expect this value to vary with time assuming the elimination rate is approximately constant. The partial derivative $\frac{\partial {T}_{1}}{\partial t}$ takes on a negative value (Scenario 4) if only if vasodilation occurs so rapidly that the second term in Eq. (5) dominates the first term. More physiologically relevant, however, is gradual vasodilation, since vessel relaxation is not instantaneous. Here, we should observe an overall increase in the value of $\frac{\partial {T}_{1}}{\partial t}$. An initial decrease, if present, arises from a positive $\frac{\partial {{\rm{v}}}_{{\rm{b}}}}{\partial {\rm{t}}}$ (i.e. active vasodilation) that reduces the effect of the first term in Eq. (5), whereas the subsequent increase in $\frac{\partial {T}_{1}}{\partial t}$ arises from a dilated blood volume fraction (i.e. a higher ${{\rm{v}}}_{{\rm{b}}}$ in the first term now dominates, especially as vasodilation slows down or ceases). Scenario 5 describes vasoconstriction. Since $\frac{\partial {{\rm{v}}}_{{\rm{b}}}}{\partial {\rm{t}}}$ is negative, the value of $\frac{\partial {T}_{1}}{\partial t}$ can only be positive. As the blood volume fraction decreases, the first term in Eq. (5) decreases, in opposition to the increase exerted by a negative $\frac{\partial {{\rm{v}}}_{{\rm{b}}}}{\partial {\rm{t}}}$ in the second term. The direction in which $\frac{\partial {T}_{1}}{\partial t}$ changes over this interval depends on the magnitude of the two opposing forces. However, once vasoconstriction has set in and blood vessels are fully constricted, the first term with a reduced ${{\rm{v}}}_{{\rm{b}}}$ dominates and there will be net decrease in the value of $\frac{\partial {T}_{1}}{\partial t}$ compared to baseline.

## Methods

### Animals and controlled gas delivery

This study was approved by the Lab Animal Services animal care committee at the Hospital for Sick Children (protocol #36668). All procedures were conducted in accordance with the Canadian Council on Animal Care. Male adult Sprague Dawley rats (*N* = 6) (Charles River Laboratories), 6 weeks old and weighing 250–300 g, were used. Rats were anesthetized on 3% isoflurane (Forene, Abbott Labs, Baar, Switzerland) in room air (2 L/min flow rate) prior to intubation with a 14-gauge angiocatheter.

A controlled gas mixing circuit described previously^7^ was used to deliver graded levels of CO_2_ and O_2_. The gas blender consisted of a computer-controlled GSM-3 mixer (CWE Inc., Ardmore, PA, USA) and blended O_2_, CO_2_, and N_2_ at a constant total flow of 2 L/min. The output gas mixture was fed first into an isoflurane vaporizer at a flow of 2 L/min and then into an MRI compatible ventilator (MRI-1 Ventilator; CWE, Ardmore, PA, USA). Mechanical ventilation was employed to allow a controlled rate and depth of breathing. The endotracheal tube was connected to a flexible tubing from the gas delivery system. Ventilation was maintained at 70 breaths per minute. Vital signs (heart rate, blood oxygen saturation) of the rat were monitored and recorded using a rodent oximeter and physiological monitor (MouseOX Plus; STARR Life Sciences) mounted on the hind paw. These physiological measurements were taken in real-time to ensure we could monitor the animal’s vital signs dynamically. A 27 G angiocath was inserted into the lateral tail vein and connected to a 3-way stopcock for contrast injection during imaging.

During MRI, animals were administered normoxia (21% O_2_ room air) to establish baseline. They were then subjected to severe hypercapnia (20% CO_2_, 21% O_2_, N_2_-balanced) followed by mild hypercapnia (5% CO_2_, 21% O_2_, N_2_-balanced).

### *In-vivo* MRI

Imaging was performed on a 3-Tesla clinical scanner (Achieva 3.0 T TX, Philips Medical Systems, best, The Netherlands), with the animal placed prone on a water blanket (HTP-1500, Adroit Medical Systems, Loudon, TN) set at 38 °C to maintain core body temperature. A receive-only 8-channel wrist coil was used for signal detection. Coronal imaging slices were positioned to include the kidneys and the leg skeletal muscle. Imaging began while animals were on a baseline period of normoxia. After 20 minutes, gadofosveset (0.3 mmol/kg) was administered via tail vein injection. A series of three-dimensional T_1_-weighted fast field echo sequences were acquired every two minutes, beginning pre-contrast during the baseline period and continuing throughout the entire examination, with the following sequence parameters: repetition time = 6.11 ms, echo time = 3.23 ms, FA = 20°, number of signal averaging = 2, field of view = 120 mm, thirteen 2-mm thick slices, and 0.6 × 0.6 mm in-plane resolution. Ten minutes after gadofosveset injection, normoxic room air was switched to 20% CO_2_, followed 16 minutes later by a switch to 5% CO_2_. A four-minute T_1_-mapping acquisition was performed prior to gadofosveset administration and repeated prior to every gas transition; the method of Cheng and Wright^11^ was used with flip angles (FA) of 2°, 10°, 25°, and 35°. Note that for presentation purposes, we show only imaging data after signal had stabilized after gadofosveset administration.

### Laser doppler perfusion measurements

To validate observations on MRI, real-time laser Doppler perfusion measurements were taken using the OxyFlo (Oxford Optronix Ltd, Oxford, UK) fiber-optic system for all animals. Measurements were in blood perfusion units (BPU), which is a relative quantity of volumetric flow rate. Note that as the catchment volume is approximately 2 mm in diameter and exceeds the spatial dimensions of an individual imaging voxel, the BPU measurement provides a perfusion index equivalent to that averaged over several voxels on MRI. A fiber-optic probe was placed in the leg muscle between the medial and lateral heads of the gastrocnemius. Experiments were conducted in the surgical suite of the animal facility instead of the MR suite to ensure sterility. Gas protocols were consistent for MRI and optical measurements.

### Data analysis

All data analysis was performed using in-house software developed in Matlab (v.8.3) (Mathworks, Natick, MA). Where *T*_1_-mapping was performed, *T*_1_ relaxation times were calculated at every pixel location across all imaging slices^11^. Regions of interest (ROIs) were outlined on these pixel-wise *T*_1_ maps, in either leg muscle or kidney cortex, to encompass the tissue at multiple contiguous slices. ROIs were tailored to the anatomy of individual animals to ensure that all relevant tissue was included. Care was taken to exclude skin, bone, and major vessels in the ROIs. The ROI-averaged *T*_1_ was then determined. To provide an impression of *T*_1_ variability within these ROIs, in leg muscle the standard deviation was within 10–15% of the mean *T*_1_, while in kidney the standard deviation was within 15–25% of the mean *T*_1_. To calculate *T*_1_-versus-time dynamics over the entire duration of the experiment, signal intensities at remaining time-points were converted to *T*_1_ values via the spoiled gradient echo signal equation, given measured pre-contrast *T*_1_ and post-contrast signal intensity. As before, an ROI-averaged *T*_1_ was calculated for all time-points. The resulting *T*_1_-versus-time curve has a temporal resolution of 2 minutes during individual gas challenge episodes and a four-minute gap where *T*_1_-mapping was acquired. Lastly, the partial derivative of *T*_1_ with respect to time was calculated at all time-points, including the 10-minute normoxic interval before switching to 20% CO_2_. A Savitzky-Golay smoothing and differentiation approach was taken to fit the *T*_1_ curve with a second-order polynomial and a 2-point window before computing the derivative. The smoothing helps to reduce the impact of variability in the data points on the computation of derivatives.

### Statistics

A one-way ANOVA was used to determine significant changes in $\frac{\partial {T}_{1}}{\partial t}$ with respect to time (i.e. twelve time-points) in the leg muscle of all six animals. A Tukey-Kramer test was used for post-hoc analysis. For optical perfusion measurements, the change in perfusion on 20% CO_2_ was calculated as the maximum difference in perfusion relative to normoxia and divided by the normoxia perfusion; the change in perfusion on 5% CO_2_ was calculated as the maximum difference in perfusion relative to 20% CO_2_ and divided by the lowest perfusion during 20% CO_2_. A student’s *t*-test was then used to determine significance in perfusion changes. For changes in heart rate, significance was determined using one-way ANOVA, followed by Tukey-Kramer post-hoc analysis, for all gas challenges. Significance is reported at a *p*-value of 5% for all ANOVA comparisons.

## Results

**Figure 1:**
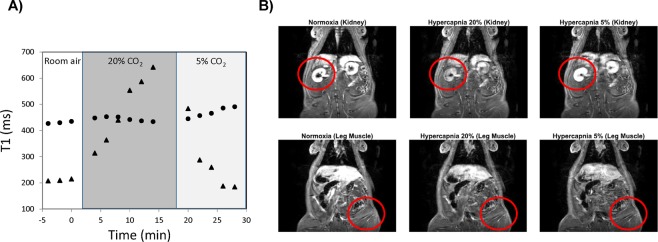
Inadequate sensitivity of *T*_1_ measurements to assess vasomodulation in low perfusion rat skeletal muscle. (**A**) The highly perfused kidney cortex (triangle) exhibits large changes in *T*_1_ in response to hypercapnia, whereas changes in skeletal muscle are very modest (circle). (**B**) Similarly, changes in signal intensity on *T*_1_-weighted images are visible in the kidney but not in the leg skeletal muscle. Red circles are provided to localize the tissues of interest.

Discerning vasomodulation is difficult in the microvascular bed of tissue with low perfusion, such as skeletal muscle. Whereas the highly perfused kidney exhibits large changes in signal intensity on T_1_-weighted images as a result of gas-induced vasomodulation, the magnitude of signal changes in skeletal muscle is much smaller. Quantitative measurement of T_1_ relaxation times did not yield a marked improvement in sensitivity to vasomodulation in muscle (Fig. 1).

**Figure 2:**
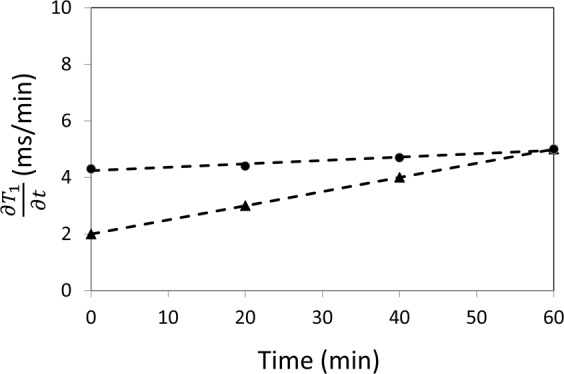
Constant renal elimination rate of contrast agent. Measurements of $\frac{\partial {T}_{1}}{\partial {\rm{t}}}$ in the absence of gas challenge (i.e. no vasomodulation) in both kidney (triangle) and skeletal muscle (circle) reveal a relatively constant rate of contrast elimination from the body. Note that this protocol is different from the gas challenge protocol used in the remainder of the study. The results shown herein support the validity of the mathematical model proposed for measuring vasomodulation in a low perfusion setting.

**Figure 3:**
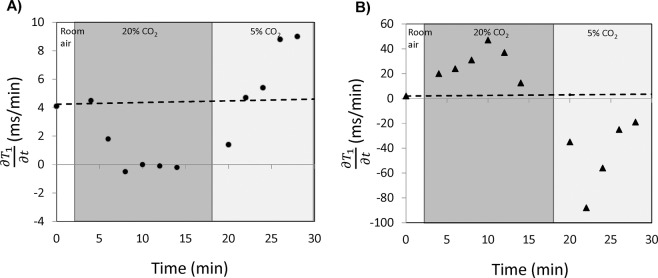
Sensitivity to vasomodulation in low perfusion setting via the parameter $\frac{\partial {T}_{1}}{\partial {\rm{t}}}$. Data for a representative rat is shown. In leg muscle (**A**), a decreasing $\frac{\partial {T}_{1}}{\partial {\rm{t}}}$ in response to 20% CO_2_ reflects a diminishing blood volume without the rapid vasoconstriction dynamics seen in the kidney cortex (**B**). Upon transitioning to 5% CO_2_, an increasing $\frac{\partial {T}_{1}}{\partial {\rm{t}}}$ reflects an expanding blood volume, again without the rapid vasodilation dynamics of kidney that results in a negative value of $\frac{\partial {T}_{1}}{\partial {\rm{t}}}$. Dashed lines representing background contrast elimination are also shown for reference.

**Figure 4:**
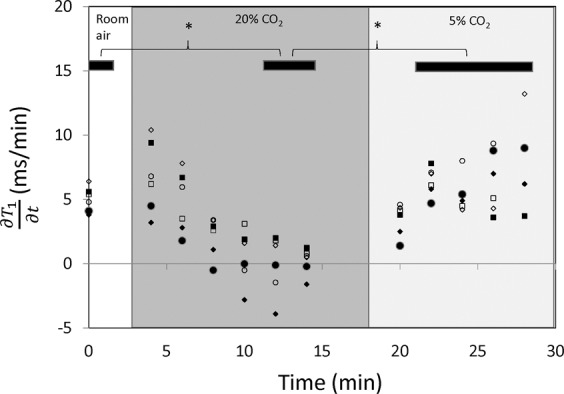
Vasomodulatory response in leg skeletal muscle across all animals. The ability to tease out vasoconstriction and vasodilation in skeletal muscle using the parameter $\frac{\partial {T}_{1}}{\partial {\rm{t}}}$ is illustrated across all animals. Note the consistency in skeletal muscle vasoreactivity to 20% CO_2_ and 5% CO_2_. The change in $\frac{\partial {T}_{1}}{\partial {\rm{t}}}$ is significantly different from baseline normoxia for the last two timepoints on 20% CO_2_; $\,\frac{\partial {T}_{1}}{\partial {\rm{t}}}$ reverses direction on 5% CO_2_, becoming significantly different by 4 minutes after gas transition (*P < 0.05). Black bars at the top of the graph indicate the data points where significance was observed.

The proposed analysis of the partial derivative $\frac{\partial {T}_{1}}{\partial t}$ dramatically increased sensitivity to vasomodulation in low perfusion tissues. Figure 2 shows the evolution of $\frac{\partial {T}_{1}}{\partial t}$ in the absence of gas challenge (i.e. no change in blood volume). A relatively flat profile is clearly evident over the 60-minute observation window, which confirms the assumption of a nearly constant rate of contrast elimination from the body. Figure 3 illustrates the evolution of $\frac{\partial {T}_{1}}{\partial t}$ in leg skeletal muscle as its microvasculature responds to gas challenge. Results in the kidney are also shown to provide a reference for interpreting the evolution of muscle $\frac{\partial {T}_{1}}{\partial t}$, as renal response to gas challenges is well understood and significant vasoconstriction and vasodilation have been consistently observed in response to 20% CO_2_ followed by 5% CO_2_^7^. The results in Fig. 2 are presented again in Fig. 3 (shown as dashed lines) to highlight vasomodulation-induced changes against a reference of background contrast elimination. The renal cortical response is consistent with our mathematical model, where positive values of $\frac{\partial {T}_{1}}{\partial t}$ during 20% CO_2_ represent vasoconstriction, with the initial increase in $\frac{\partial {T}_{1}}{\partial t}$ depicting the effect of active vasoconstriction and the later decrease depicting a slowing down or plateauing of this process. After the switch to 5% CO_2_, $\frac{\partial {T}_{1}}{\partial t}$ becomes negative as a result of rapid vasodilation. In leg skeletal muscle, the trends observed suggest gradual vasoconstriction ($\frac{\partial {T}_{1}}{\partial t}$ remains positive but decreases or stabilizes). In some animals, as seen later in Fig. 4, $\frac{\partial {T}_{1}}{\partial t}$ initially spikes above baseline value, indicating immediate vasoconstriction. Upon transition to 5% CO_2_, rapid vasodilation is absent (otherwise, $\frac{\partial {T}_{1}}{\partial t}$ would become negative as in the kidney), but $\frac{\partial {T}_{1}}{\partial t}$ continues to increase, reflecting sustained vasodilation on 5% CO_2_. Figure 4 summarizes the vasomodulatory responses in the leg muscle of all animals. The overall trend in $\frac{\partial {T}_{1}}{\partial t}$ changes was consistent, supporting the robustness of our new method. The decrease in $\frac{\partial {T}_{1}}{\partial t}$ in response to 20% CO_2_ is significant after 10 minutes on hypercapnia (absolute time is 12 minutes), and the overall increase upon transitioning to 5% CO_2_ becomes significant by 4 minutes post-gas transition (absolute time is 22 minutes). Note that in a few animals, $\frac{\partial {T}_{1}}{\partial t}$ becomes negative towards the end of 20% CO_2_. This reflects an increase in blood volume from perfusion pressure as blood is pushed out of the highly vasoconstricted kidney upstream of the leg muscle.

**Figure 5:**
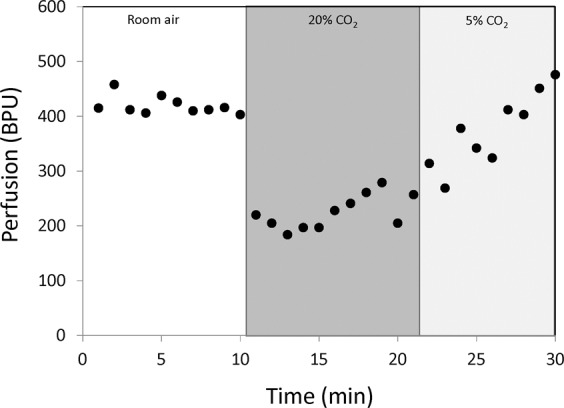
Optical perfusion validation in leg muscle. Laser Doppler perfusion measurements over the course of hypercapnic challenge intervals are shown for a representative rat. Blood perfusion units (BPU) are relative quantities and do not indicate absolute blood flow.

**Figure 6:**
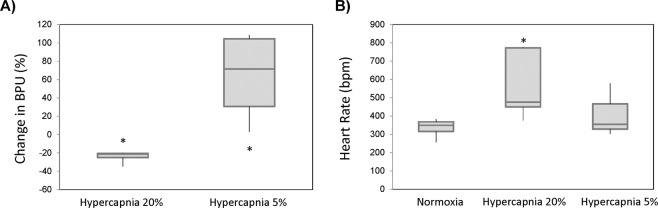
Average changes in perfusion and heart rate in response to severe and mild hypercapnia. (**A**) The maximum perfusion change in leg skeletal muscle induced by 20% CO_2_ as measured on laser Doppler and the maximum vasodilation induced by 5% CO_2_ are averaged over all animals. (**B**) The average heart rates during normoxia, 20% CO_2_, and 5% CO_2_ are also shown. Shown are the median, first and third quartile values, and minima and maxima across all animals (*P < 0.05).

Figure 5 illustrates a laser Doppler perfusion recording in a representative rat. During the severe hypercapnia interval, perfusion in leg muscle is seen to decrease immediately followed by a gradual increase, which continues upon transition to 5% CO_2_. Figure 6 summarizes the perfusion response across all animals, and the trend is in agreement with MRI: vasoconstriction on 20% CO_2_ and vasodilation on 5% CO_2_. Note, however, that it is difficult to compare the amplitude of response between optical perfusion measurements and MRI measures of vasomodulation. This discrepancy arises from the fact that these two metrics are, in fact, different: the MRI metric is robust against the heart rate variation to which optical perfusion is susceptible. As seen in Fig. 6B, the heart rate increases during 20% CO_2_ and decreases during 5% CO_2_; these changes counter both the reduction in flow from vasoconstriction during 20% CO_2_ and the increase in flow from vasodilation during 5% CO_2_. In fact, the observed increased perfusion on optical measurements during the latter phase of 20% CO_2_ may be due largely to a much higher heart rate rather than active vasodilation. Therefore, both panels of Fig. 6 must be interpreted together when comparing against MRI measurements.

## Discussion

The skeletal muscle microvasculature is involved in numerous pathologies, including ischemia and metabolic syndromes such as diabetes. A technology that opens a window on this system would be invaluable for the early detection of various diseases. Currently, no technology exists to probe the microvasculature non-invasively in deep, low perfused tissue. Specifically, there is no technology to measure vasomodulatory capacity, or vasoreactivity, which is an important index of vascular health and reflects the ability of microvessels to adjust blood flow in response to changing metabolic demands. This paper describes a new analytical method that allows sensitive assessment of microvascular vasomodulation in low perfusion tissue. Compared to assessment of *T*_1_ changes in response to hypercapnia, which have been shown in the literature to be insignificant in skeletal muscle^7^, changes in $\frac{\partial {T}_{1}}{\partial t}$ are significant. In this study, we showed that the metric $\frac{\partial {T}_{1}}{\partial t}$ was sensitive to both vasoconstriction and vasodilation in the leg skeletal muscle, and MRI results were corroborated by invasive Doppler perfusion measurements. Of note is that this new metric works in healthy tissue of normal animals, as demonstrated in this study, and can distinguish unhealthy microvasculature in an injury setting^12^.

When interpreting the temporal evolution of the new metric $\frac{\partial {T}_{1}}{\partial t}$, one must bear in mind that two discrete influences determine the value of $\frac{\partial {T}_{1}}{\partial t}$: the absolute microvascular blood volume at any point in time and the active vasomodulation at that instant in time. MRI results in leg skeletal muscle across all animals revealed a consistent initial decrease in $\frac{\partial {T}_{1}}{\partial t}$ immediately after exposure to 20% CO_2_ – this decrease is due primarily to an overall reduction in blood volume from vasoconstriction. Halfway through this hypercapnic interval, $\frac{\partial {T}_{1}}{\partial t}$ plateaus at a level much lower than normoxic baseline; optical perfusion measurements confirmed a return to increased perfusion at this time. The most likely explanation for this reversal phenomenon is a cessation in the constriction of blood vessels as a result of higher pressure from a greatly elevated heart rate and from blood redistribution arising from renal vasoconstriction. On transition to 5% CO_2_, $\frac{\partial {T}_{1}}{\partial t}$ continues increasing as blood vessels dilate to accommodate higher perfusion in the presence of a lowered heart rate.

In the kidney, vasoconstriction was also observed during 20% CO_2_ followed by vasodilation during 5% CO_2_. However, the evolution of $\frac{\partial {T}_{1}}{\partial t}$ exhibits a few notable differences. First, upon application of 20% CO_2_, several animals demonstrated an initial “hump” in $\frac{\partial {T}_{1}}{\partial t}$. This is most likely a result of active vasoconstriction (second term in Eq. (5)) dominating the influence of a total lower blood volume (first term in Eq. (5)). Similarly, during the initial interval after the transition to 5% CO_2_, the values of $\frac{\partial {T}_{1}}{\partial t}$ were negative, which implies active vasodilation to the extent that, again, the second term in Eq. (5) dominates. Another interesting observation is that as renal vasodilation continues during the 5% CO_2_ interval, a point is reached where blood is diverted *from* skeletal muscle downstream from the kidney – this is reflected in the plateau/downturn of the $\frac{\partial {T}_{1}}{\partial t}$ graph in leg muscle in Fig. 4 and is known as the steal effect.

It is worth noting that a truly equivalent validation method does not exist. In this study, we used optical Doppler perfusion measurements as the best alternative, but like most clinical techniques for assessment of vasomodulation, optical Doppler also evaluates volumetric blood flow and not changes in vessel tone. An increase in perfusion measured on optical Doppler can simply result from a higher heart rate without necessarily changes in vessel diameter. However, when we take in account heart rate variations in our interpretation of Doppler perfusion measurements, we can form a more accurate understanding of vasomodulatory responses. For example, during 20% CO_2_, Doppler measurements indicated decreased perfusion at the same time that heart rate was greatly elevated. In the presence of greater perfusion pressure, decreased perfusion can only arise as a result of vasoconstriction.

Our model is a first-order mathematical approximation of the effect of vasomodulation on contrast-induced *T*_1_ effects. It assumes that renal elimination of the contrast agent is constant, but this may be true only over a short time interval and not over the entire duration of the imaging experiment. Future work will model more accurately this elimination profile. The model also is not sensitive to second-order effects that may distinguish more clearly rapid versus slow vasomodulation. In its present form, our model can clearly separate vasodilation from vasoconstriction but cannot ascribe the change to hormonal or neuronal factors^13,14^ on the basis of the timing of vasomodulation. Further efforts are required to develop a more complex model that encompasses these elements.

There are several points to consider when implementing the proposed method for assessing microvascular reactivity. First, the measurement duration must be short with respect to the phenomenon of interest. In other words, we must achieve a sufficiently high temporal resolution in our MR acquisition to capture the dynamics of a changing blood volume. Given that vasomodulation occurs on the time scale of minutes, we are well within the required sampling rate. However, while very rapid imaging is unnecessary, it would be beneficial to increase the temporal resolution beyond the 2-minute interval in our current implementation. Doing so would increase the robustness of analyzing the first-order differential of *T*_1_ versus time, as decreased temporal spacing between data-points would improve the accuracy of derivative estimates. It is important to note that the value of $\frac{\partial {T}_{1}}{\partial t}$ on either side of a gas transition will have contributions from before or after the transition; this inevitable “artifact” means that the calculated $\frac{\partial {T}_{1}}{\partial t}$ values immediately adjacent to a gas transition should be interpreted with caution. With our current temporal sampling every 2 minutes, excluding these transitions points may remove important information. However, if our data had been acquired at a higher temporal resolution, we could have easily eliminated $\frac{\partial {T}_{1}}{\partial t}$ values on either side of a gas transition without sacrificing the time extent over which we characterize $\frac{\partial {T}_{1}}{\partial t}$ changes. Another opportunity also arises when the acquisition speed is increased: the proposed analysis approach may be applied on a higher temporal resolution dataset to assess phenomena with rapid dynamics, such as spontaneous vasomotion and pulsatility. The last point to consider is water exchange, which may occur between the intra- and extra-vascular compartments and exert an influence on *T*_1_ measurement. However, water exchange between these compartments has been shown to reside predominantly in the slow regime^15^, and the effect on *T*_1_ would be minimal.

A final comment pertains to the study of microvascular dynamics. There is ample literature on the oscillatory nature of microvascular blood flow, with the majority employing optical perfusion methods with a very high temporal sampling rate^16,17^. With such rapid sampling, one can observe a wide frequency range associated with cardiac, respiratory, or myogenic origins. Sophisticated extraction of these distinct frequency components is possible through wavelet analysis and non-linear mode decomposition^17^. These analysis methods may potentially be valuable for analyzing our MRI measurements; to do so, we would need to increase our temporal resolution to exceed at minimum 10 seconds and ideally approach 1 second.

The ultimate utility of the proposed analytical method will rest on its ability to identify diseased or injured microvessels on the basis of their vasomodulatory capacity. There are a number of models to investigate, including ischemic insult, hypertension, and obesity. In ischemic injury where the endothelium is affected, we can expect a reduced capacity to adjust vessel tone compared to healthy microvessels. Future work will focus on a range of microvascular pathologies to validate the general utility of the new method in skeletal muscular disease, cerebrovascular disease, and other pathologies in a low perfusion setting.

There is finally the consideration of eventual translation to humans. The contrast agent employed, gadofosveset, had been FDA and Health Canada approved and was used clinically for several years for MR angiography and cardiac MR exams until it was recently withdrawn due to profitability, not safety, reasons. However, if additional indications that justify the need for blood-pool agents are put forth, the reinstatement of these agents into the clinic is highly probable. Given that all other technical aspects, including the gas challenges, have been shown to be safe in humans, the translational path for the proposed technology is viable.

## Conclusions

We have presented a novel analytical method to assess microvascular vasomodulation, or vasoreactivity, in low perfusion tissue. To our best knowledge, this is the only non-invasive, deep-tissue imaging technique of its kind. We have demonstrated the capabilities of this technique in skeletal muscle, which has very low perfusion relative to other organs. The method can be applied to other modestly perfused tissues to investigate pathologies where microvascular dysfunction is an early biomarker of disease or injury.

## Data Availability

The datasets generated during and/or analysed during the current study are available from the corresponding author on reasonable request.
